# Understanding intersections of social determinants of maternal healthcare utilization in Uttar Pradesh, India

**DOI:** 10.1371/journal.pone.0204810

**Published:** 2018-10-04

**Authors:** Arnab Dey, Katherine Hay, Bilal Afroz, Dharmendra Chandurkar, Kultar Singh, Nabamallika Dehingia, Anita Raj, Jay G. Silverman

**Affiliations:** 1 Sambodhi Research and Communications Pvt. Ltd., Noida, India; 2 Bill and Melinda Gates Foundation, Seattle, Washington, United States of America; 3 Center on Gender Equity and Health, Division of Global Public Health, University of California, San Diego School of Medicine, La Jolla, California, United States of America; The University of Warwick, UNITED KINGDOM

## Abstract

**Objective:**

To explore intersections of social determinants of maternal healthcare utilization using the Classification and Regression Trees (CART) algorithm which is a machine-learning method used to construct prediction models.

**Methods:**

Institutional review board approval for this study was granted from Public Health Service—Ethical Review Board (PHS-ERB) and from the Health Ministry Screening Committee (HMSC) facilitated by Indian Council for Medical Research (ICMR). IRB review and approval for the current analyses was obtained from University of California, San Diego. Cross-sectional data were collected from women with children aged 0–11 months (n = 5,565) from rural households in 25 districts of Uttar Pradesh, India. Participants were surveyed on maternal healthcare utilization including registration of pregnancy (model-1), receipt of antenatal care (ANC) during pregnancy (model-2), and delivery at health facilities (model -3). Social determinants of health including wealth, social group, literacy, religion, and early age at marriage were captured during the survey. The Classification and Regression Tree (CART) algorithm was used to explore intersections of social determinants of healthcare utilization.

**Results:**

CART analyses highlight the intersections, particularly of wealth and literacy, in maternal healthcare utilization in Uttar Pradesh. Model-1 documents that women who are poorer, illiterate and Muslim are less likely to have their pregnancies registered (71.4% vs. 86.0% in the overall sample). Model-2 documents that poorer, illiterate women had the lowest ANC coverage (37.7% vs 45% in the overall sample). Model-3, developed for deliveries at health facilities, highlighted that illiterate and poor women have the lowest representation among facility deliveries (59.6% vs. 69% in the overall sample).

**Conclusion:**

This paper explores the interactions between determinants of maternal healthcare utilization indicators. The findings in this paper highlights that the interaction of wealth and literacy can play a very strong role in accentuating or diminishing healthcare utilization among women. The study also reveals that religion and women’s age at marriage also interact with wealth and literacy to create substantial disparities in utilization. The study provides insights into the effect of intersections of determinants, and highlights the importance of using a more nuanced understanding of the impact of co-occurring forms of marginalization to effectively tackle inequities in healthcare utilization.

## Introduction

The Sustainable Development Goals for 2030 continue the focus on maternal health, pushing beyond the targets set by the Millennium Development Goals for 2015. The SDGs call for reducing maternal mortality across the globe to less than 70 per 100,000 live births by the year 2030. This goal also highlights the immense disparities between developing and developed regions in their contributions to the global maternal mortality ratio [1]. Globally, 99% of all maternal deaths are estimated to occur in developing regions [2]. India, with a Maternal Mortality Ratio of 167 [3] contributed nearly 15 percent of global maternal deaths in 2015 [2]. In addition to huge disparities existing between developed and developing regions, maternal mortality is disproportionately high among socially and economically marginalized communities within regions[4, 5].

It is well established that the majority of maternal deaths may be prevented by providing basic maternal health services to women [6]. However, social and economic disparities in utilization of such basic health services also exist [7]. Multiple studies have shown that women belonging to marginalized social strata have poorer utilization of healthcare services [8, 9]. Studies have also shown that women’s literacy status, household wealth and age are crucial determinants of utilization [7, 10, 11]. Other studies have indicated that social group (e.g., caste), religion and women’s age at marriage also play a crucial role in determining utilization of healthcare services [12].

While inequities in healthcare utilization have been well-described, most explorations have been limited to uni-dimensional analyses [13, 14]. However, several studies have found that the interaction of social determinants at a micro level creates significant differences in healthcare utilization.

Iyer et. al., in their review of research on the effects of intersections between gender and class on health status and access to health, found that intersections have the potential to significantly alter the impacts as compared to any one dimension of inequality considered alone. Their research suggests that the study of any one dimension can lead to misinterpretation of differences and has a significant human cost [15]. Weber and Parra-Medina also acknowledge the significance of intersectional approaches in charting a path to eliminate health disparities [16]. Östlin et. al., in their paper on priorities for research on equity and health, stress the need to examine *intersections* among social determinants of health status and behaviors [17]. Understanding such intersections can help maximize the efficiency of programs promoting healthcare utilization by facilitating more precise targeting of sub-groups in greatest need.

The understanding of intersections of social determinants of healthcare utilization is still evolving [18] and is limited by inadequacies of methods to quantitatively understand the intersections at play [14, 16]. In this paper, we use the Classification and Regression Tree (CART) approach [19] to identify population segments defined by multiple forms of marginalization to specify the full range of differences in healthcare utilization across the demographic spectrum. We hypothesize that intersections of social determinants are associated with significant differences in healthcare utilization by women. Specifically, we explore intersections of wealth, social group, religion, age, and age at marriage in relation to maternal healthcare utilization in Uttar Pradesh. Uttar Pradesh is the most populous state in India, and has a maternal mortality ratio of 285 deaths per 100000 live births, which is much higher than the national average of 167 [3]. Maternal healthcare utilization outcomes in this study include registration of pregnancy, receipt of any antenatal care (ANC) in the third trimester of pregnancy, and delivery at a health facility.

## Methods

Institutional review board approval for this study was granted from Public Health Service—Ethical Review Board (PHS-ERB) and from the Health Ministry Screening Committee (HMSC) facilitated by Indian Council for Medical Research (ICMR). IRB review and approval for the current analyses was obtained from University of California, San Diego.

Data for the current study were obtained as a part of a midline evaluation of the Uttar Pradesh Technical Support Unit (UP-TSU; a set of interventions to improve the reach and quality of public health services) conducted between June and October 2016. Rural households from the 25 intervention districts of Uttar Pradesh were considered for the study. The intervention districts were chosen because they had the highest rates of maternal and infant mortality and other critical health and development indicators [7]. Across the 25 districts, a multistage sampling design was used to create a representative sample of women with live births in the last 12 months. A census of all households was first undertaken in the Primary Sampling Units (PSUs) to identify all women who had a live birth in the last 12 months. Overall, 19,951 live births (in the past 12 months) were identified from 900 PSUs, of which 7,200 live births were randomly sampled for the study. All sampled women were approached to participate in the study, and 5,565 women consented to participate (77% participation rate). Further details of the study design are described by Seth et. al. [7].

The study was conducted by female research assistants, trained in maternal and child health outcomes, health systems and survey research. All participants agreeing to participate in the study provided informed written consent prior to the survey. Participants were not given any monetary incentive to participate in the study. All participants were interviewed in a private setting with the interviews lasting around 60 minutes. Data were collected on mobile handheld devices and included no individual identifiers. The collected data were uploaded to a secure data management system for analysis.

The study was approved by the National Rural Health Mission (NRHM), and by the Public Health Service—Ethical Review Board (PHS-ERB). Health Ministry Screening Committee (HMSC) approval, facilitated by Indian Council for Medical Research (ICMR), was also obtained.

### Measures

#### Dependent variables

Three key outcomes related to care during pregnancy were considered: registration of pregnancy (ensuring inclusion in the public health system), receipt of any antenatal care in the third trimester, and delivery at a health facility (public or private). Registration of pregnancy was chosen as it is the first point of contact the public health system for pregnant women and ensures their inclusion among those eligible to receive health services offered by the state. Receipt of antenatal care in the third trimester was also chosen as a dependent variable, as antenatal care checkups in the third trimester are vital to identify any complications during pregnancy. Timely identification of such complications facilitates appropriate referral and management during delivery. Finally, delivery at a health facility was also chosen, as it implies delivery by Skilled Birth Attendants, which plays a crucial role in managing complications during the intra-partum and post-partum periods, reducing maternal and neonatal mortality. All three outcomes were considered as dichotomous variables.

#### Independent variables

Known socio-demographic indicators of health inequities and factors known to affect healthcare utilization were included as independent variables in the model. The selected variables included social group, religion, household wealth, women’s literacy status, age of women, and age at marriage. The study was conducted in rural areas of Uttar Pradesh. Since all the women in the sample belonged to rural areas, the place of residence of participants (rural / urban) was not included as a determinant. All measures were assessed using core items from the Demographic and Health Surveys (DHS) conducted in India [20]. Households were classified into three categories based on their social group to indicate marginalization: Scheduled Caste / Scheduled Tribe (most marginalized), Other Backward Classes (OBC) and Others (least marginalized). A dichotomous measure was created to classify religion of households as Muslim or non-Muslim. To focus on the most prevalent form of religion-based marginalization, the small proportion of households that were neither Hindu nor Muslim were classified as non-Muslim. Economic status of participants was assessed via the Wealth Index, which is a continuous measure of household wealth and is used in the DHS in India [21]. The wealth index was divided into 5 equal quintiles to classify households as either lowest, low, medium, high and highest wealth strata. Each stratum comprised 20% of sample households. Women’s literacy status was measured as a dichotomous variable to capture whether respondents could read and write. In addition, age of participants at the time of interview and their age at their first marriage were treated as continuous variables.

### Data analysis

The Classification and Regression Tree (CART) algorithm, introduced by Breiman et. al. [19], was used for the current analysis. CART is a non-parametric statistical approach that uses the recursive partitioning technique to split a sample population into sub-groups based on a pre-defined criteria. When provided with a dependent variable (either categorical or continuous) and a set of independent demographic variables hypothesized to influence the dependent variable, CART creates mutually exclusive sub-groups based on combinations of demographics within the sample and the proportion of individuals in a particular sub-group who are likely to engage in the behaviour / practice represented by the dependent variable [22].

At the first stage, for a particular dependent variable, the CART model will first begin the procedure with the entire sample as the first group or “parent node”. From the parent node, the model then begins to recursively split the sample into binary sub-groups (called child-nodes) based on the subsequent stepwise addition of individual independent variables. To select the independent variable for the split at any stage, the model lines up all the independent variables and selects the variable that creates the two most disparate sub-groups in terms of the dependent variable. Once the independent variable to be used to split the sample in a particular step has been chosen, the model then decides the cut-off point for the chosen independent variable at which the group would be split into sub-groups. This decision is made based on a splitting criterion—which is a function of the degree of impurity of a child node. Impurity of a node can be generally described as the variability within the node in terms of the dependent variable. For example, a node that has all the individuals under one category of a dichotomous dependent variable (all 0s or all 1s) will be a perfectly pure node [22]. Similarly, a node will have the maximum impurity when there are equal number of individuals in the two categories of the dependent variable (equal number of 0s and 1s). Measures of impurity include: *Entropy*, *Gini*, *and Classification error* [23], with the Gini improvement measure being most commonly used for deciding the splitting criteria. Lemon et. al. elucidate the steps involved in estimating the Gini improvement measure [22]. For the current study, we use the Gini improvement measure to determine the cut-off points.

This process of identifying and adding independent variables based on a decided cut-off continues until a stopping condition is satisfied. The CART model allows users to specify a stopping condition to terminate the process of recursive partitioning. There are a number of different criteria that may be specified in the stopping condition. For example, specifying the value of impurity measure in a node as a threshold, specifying a minimum number of individuals in a child node, specifying the maximum number of iterations that a model can have etc. For the current study, the stopping criterion was based on multiplicity adjusted p-values, and the minimum criteria in order to split a node was set at p = 0.95 i.e. the p-value had to be less than 0.05 for a node to split further. Three separate models were developed based on each of the dependent variables. The models were developed using RStudio (version 1.0.136) software.

## Results

Participants included 5,565 women with children aged 0–11 months. The mean age of participants was 26.4 years (SD = 4.4) and their mean age at first marriage was 18.1 years (SD = 2.4). Approximately half of participants were illiterate (53.8%) and the majority belonged to either the SC/ST (29.3%) or the Other Backward Classes (OBC) category (53.5%). Overall, 16.2% of the participants were Muslims and almost half of the participants had 3 or more births at the time of the interview (45.9%). These proportions closely correspond to the Census of India 2011 figures for rural Uttar Pradesh. According to the Census, 54.9% of rural women in Uttar Pradesh were illiterate, 23.6% of rural women belonged to the SC/ST social groups and 19.3% of the women in the state were Muslims [24, 25].

Overall 85.5% of the contacted women had registered their pregnancy, 45.2% of the women received any antenatal care in the last trimester of their pregnancy, and 69.4% of the participants had delivered at a health facility. Differences in the proportion of women reporting each outcome varied significantly based on all social determinants considered, with the exception of women’s religion and receipt of ANC in the third trimester (Table 1).

**Table 1:** Healthcare utilization by women with children aged 0–11 months from 25 high priority districts in Uttar Pradesh (N = 5,565) across social determinants of health.

	Total Sample	Pregnancy registration		Any ANC in the third trimester		Institutional Delivery	
	(n)	% (n)	p-value*	% (n)	p	% (n)	P
**Social Groups**							
SC/ST	29.3 (1629)	84.5 (1376)	<0.01	41.6 (678)	<0.01	66.2 (1078)	<0.01
OBC	53.5 (2977)	87.3 (2600)		45.3 (1348)		69.6 (2073)	
Others	17.2 (959)	82.1 (787)		51.0 (489)		73.9 (709)	
**Religion**							
Muslim	16.2 (901)	81.7 (736)	<0.01	46.2 (416)	0.52	65.5 (590)	<0.01
Non-Muslim	83.8 (4664)	86.3 (4027)		45.0 (2099)		70.1 (3270)	
**Wealth**							
Lowest	20.0 (1113)	79.6 (886)	<0.01	38.4 (427)	<0.01	63.8 (710)	<0.01
Low	20.0 (1113)	81.9 (912)		39.4 (439)		60.3 (671)	
Medium	20.0 (1113)	88.0 (979)		42.3 (471)		67.0 (746)	
High	20.0 (1113)	89.4 (995)		47.2 (525)		74.7 (831)	
Highest	20.0 (1113)	89.0 (991)		58.7 (653)		81.0 (902)	
**Literacy**							
Illiterate	53.8 (2996)	83.1 (2489)	<0.01	38.6 (1156)	<0.01	62.1 (1860)	<0.01
Literate	46.2 (2569)	88.5 (2274)		52.9 (1359)		77.9 (2000)	
**Age**							
Below 20 years	58	86.2(50)	<0.01	48.3(28)	<0.01	72.4(42)	<0.01
20–24 years	1814	88.3(1601)		49.1(890)		76.4(1385)	
25–29 years	2287	85.4(1954)		45.9(1049)		67.5(1543)	
30–34 years	946	82(776)		39.5(374)		63.9(604)	
35 years or above	460	83(382)		37.8(174)		62.2(286)	
Mean	18.1	18.2	<0.01	18.3	<0.01	18.3	<0.01
Std. deviation	2.4	2.4		2.4		2.4	
**Age at Marriage**							
Age at marriage (< = 16 yrs)	1123	83.6(939)	<0.04	41.0(461)	<0.01	61.8(695)	<0.01
Age at marriage (>16 yrs)	4442	86.0(3824)		46.2(2054)		71.2(3165)	
Mean	18.1	18.2	<0.01	18.3	<0.01	18.3	<0.01
Std. deviation	2.4	2.34		2.4		2.4	

*p-values assess differences between groups who utilized the service on the given variable, based on chi-square analyses for categorical variables and t-tests for continuous variables.

We refer to the terminology of decision trees to explain the results. The first splitting variable in the tree is referred as the root node and all subsequent splitting variables are referred as internal nodes. Each node splits the sample in two sub-groups which are known as branches. The last nodes in the tree after which the splitting stops are called leaf nodes [23].

**Fig 1:**
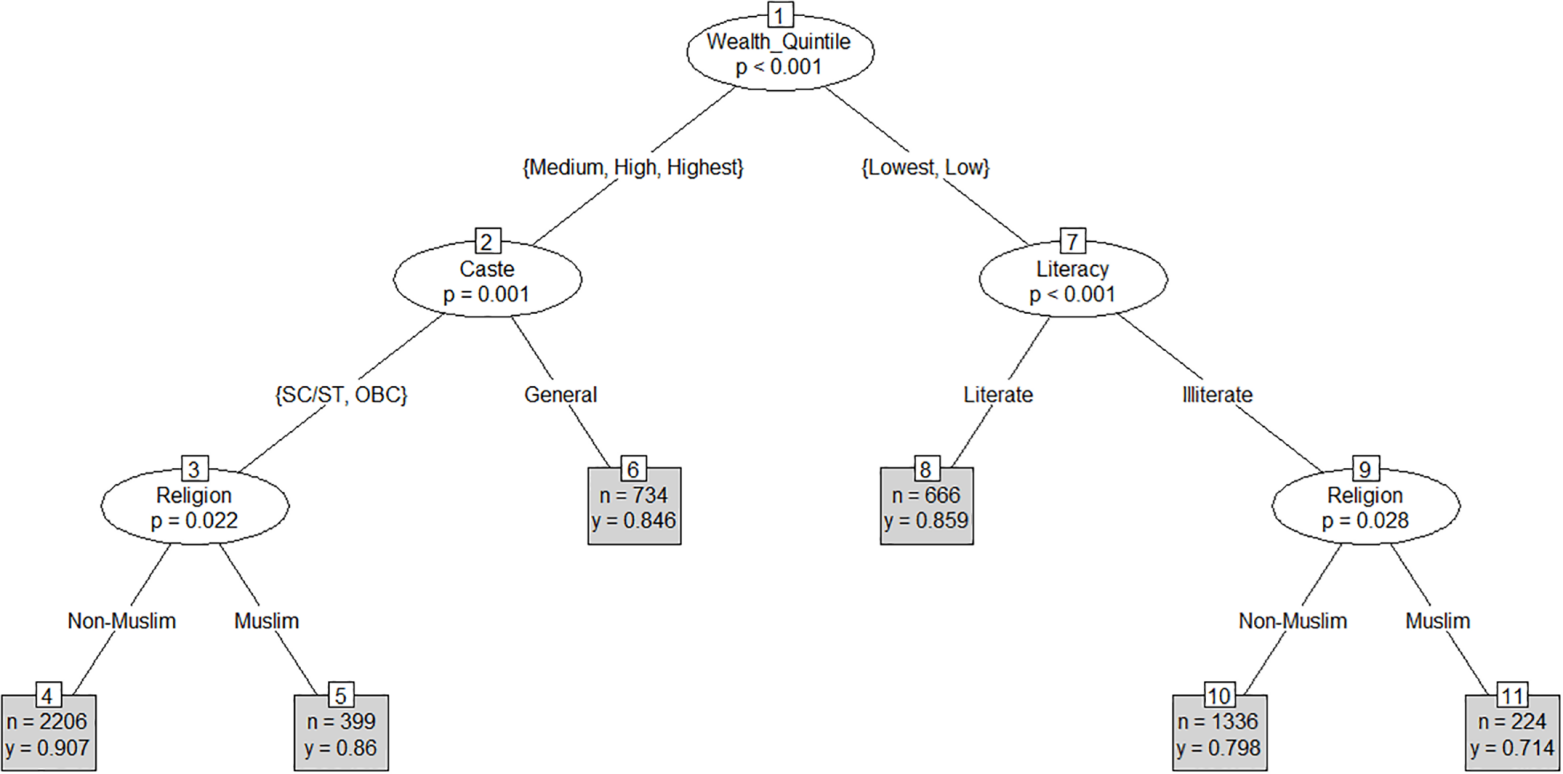
CART model for pregnancy registration (Mean = 0.86).

In case of pregnancy registration, the CART model identified wealth as the root node (node 1), which split the sample into two branches of higher and lower household wealth (p<0.001) (Fig 1). The model then splits the branch of higher wealth quintile by social group (p = 0.001) (node 2). The first branch from node 2 comprises households belonging to the marginalized social groups, i.e. Scheduled Castes, Scheduled Tribes (SC/ST) and Other Backward Classes (OBCs). The second branch from node 2 comprises of households belonging to the non-marginalized social groups (others). This branch leads to a leaf node (node 6), while the branch comprised of the marginalized social groups is further split into two branches by religion (node 3)—Muslim and Non-Muslim (p = 0.022). The branch of lower wealth quintile is split by literacy (node 7)–literate and Illiterate (p<0.001). While the splitting stopped at leaf node 8, the branch of illiterate women belonging to the lower wealth quintiles (low or lowest) was further split by religion (node-9) (p = 0.028)–Muslim and Non-Muslim.

The model shows that 90.7% (n = 2,737) of non-Muslim participants belonging to marginalized social groups (SC/ST/OBCs) and higher wealth quintiles (medium, high or highest) had registered their pregnancies vs 86% (n = 399) of Muslim participants with similar household wealth. Further, 85.9% (n = 666) of literate participants belonging to the lower wealth quintiles (low or lowest) had registered their pregnancies. Among illiterate Muslim participants belonging to the lower wealth quintiles (low or lowest), 71.4% (n = 224) had registered their pregnancies as compared to 79.8% (n = 1,336) of illiterate non-Muslim participants of similar household wealth.

Overall, the highest difference in pregnancy registration was found between leaf node 4 representing non-Muslim women belonging to marginalized social groups and higher wealth quintiles (90.7%) vs leaf node 11 representing Muslim and illiterate women belonging to the lower wealth quintiles (71.4%).

**Fig 2:**
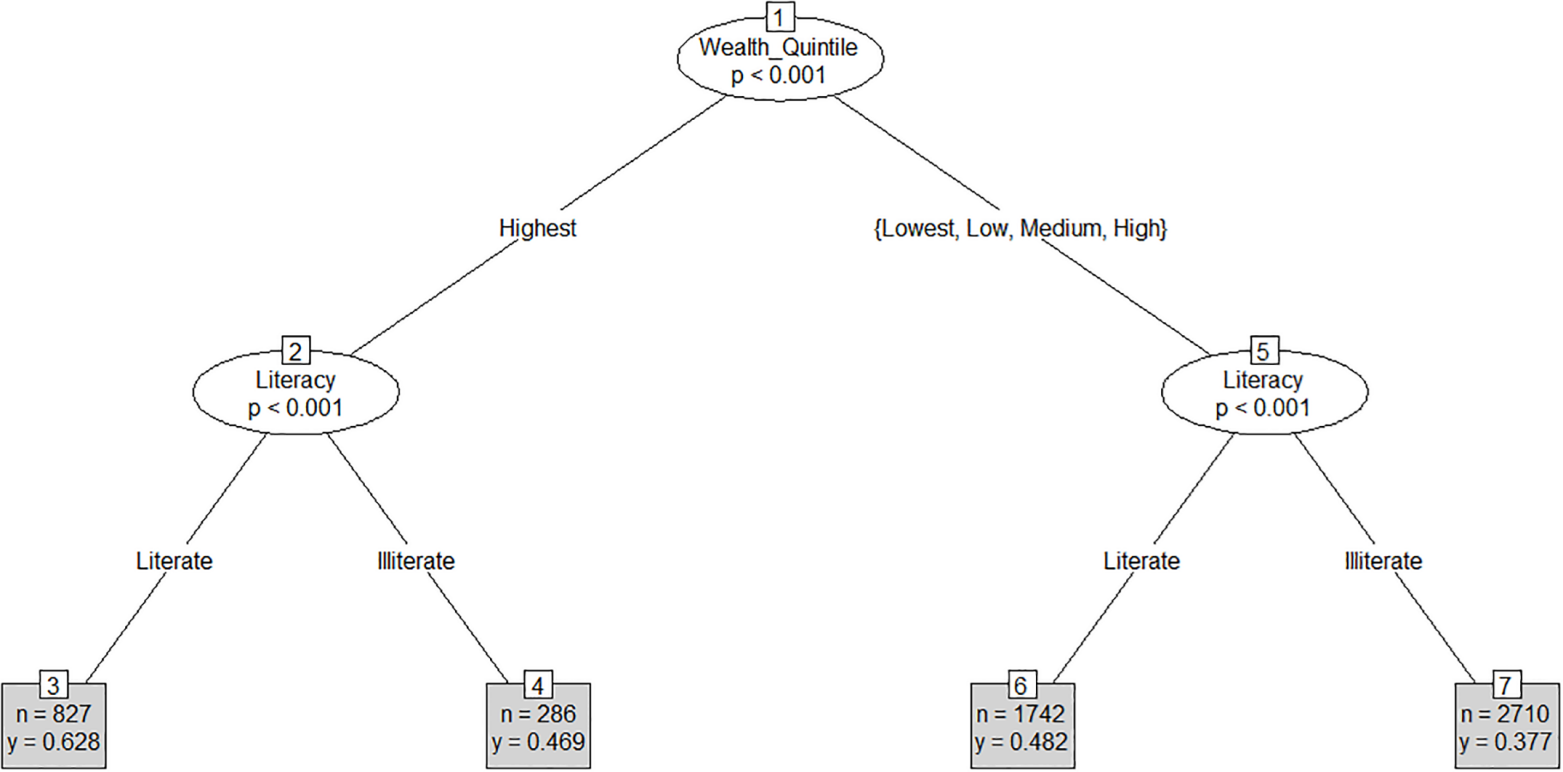
CART model for receipt of any ANC in the 3rd trimester (Mean = 0.45).

For the outcome of receipt of any ANC in the last trimester of pregnancy, the CART model identified wealth as the root node (node 1), which split the sample into two branches of highest and lower (high, medium, low or lowest) household wealth (p<0.001) (Fig 2). The model then split the branch of highest wealth quintile by literacy (node 2)–Literate and Illiterate (p<0.001) and the branch of lower wealth quintiles by literacy (node-5)–Literate and Illiterate (p<0.001). The splitting stopped at leaf nodes 3, 4, 6, and 7. The model shows that 62.8% (n = 827) of literate participants belonging to highest wealth quintile received any ANC in the last trimester during pregnancy vs 46.9% (n = 286) of illiterate participants belonging to the highest wealth quintiles. Further, 48.2% (n = 1,742) of literate participants belonging to lower wealth quintiles (high, medium, low or lowest) received any ANC in the last trimester vs. 37.7% (n = 2,710) of illiterate participants belonging to lower wealth quintiles (high, medium, low or lowest).

Overall, the greatest disparity in receipt of any ANC in the last trimester was found between leaf node 3, representing literate women belonging to the highest wealth quintile (62.8%), and leaf node 7, representing illiterate women belonging to the bottom four wealth quintiles (37.7%).

**Fig 3:**
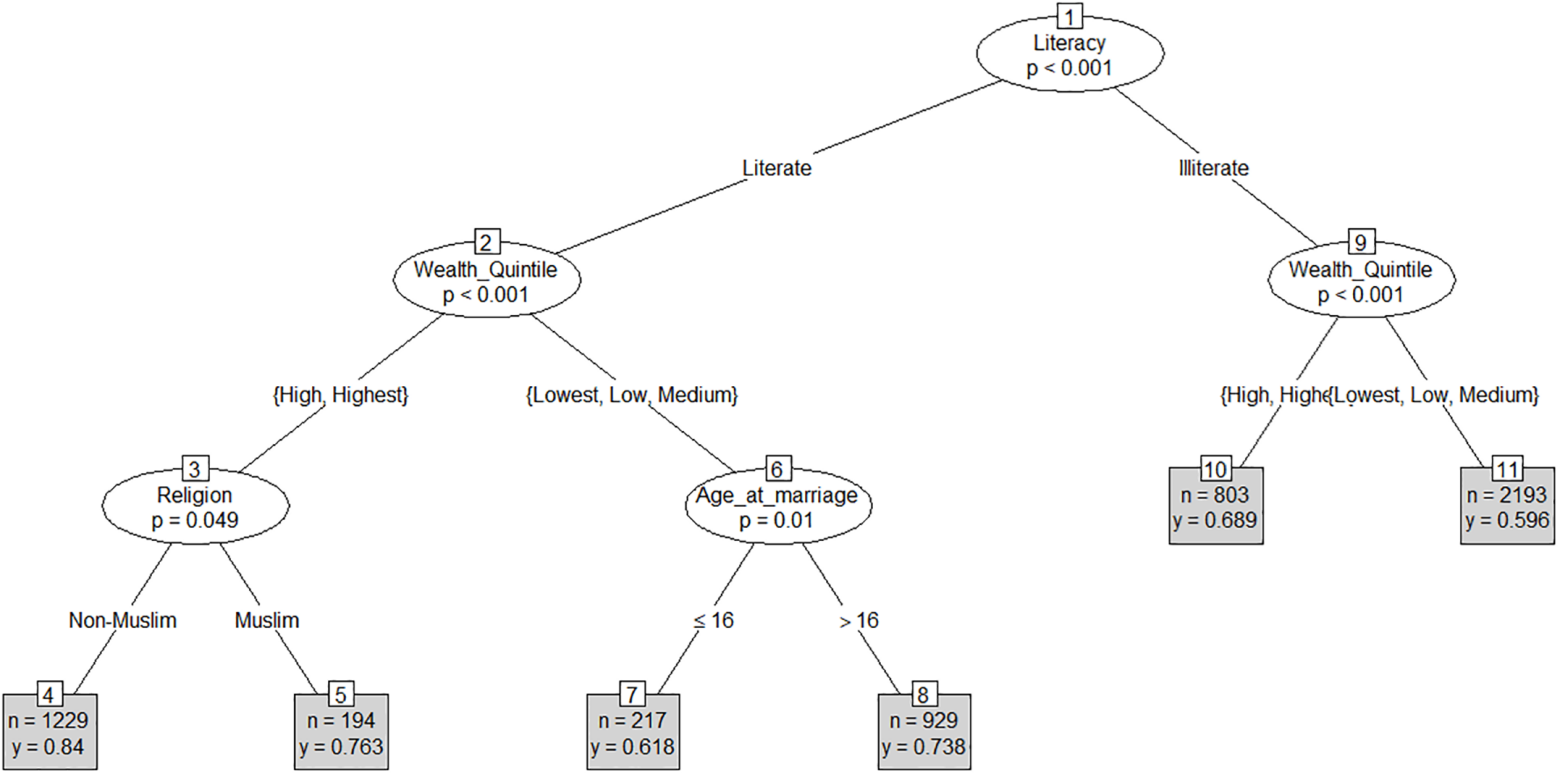
CART model for delivery at health facilities (Mean = 0.69).

In case of women opting to deliver at a health facility, the CART model identified literacy as the root node (node 1), which split the sample into two branches based on literacy (p<0.001) (Fig 3). The model then split the branches of both literate and illiterate participants by wealth quintiles—the top two quintiles (high and highest) and the bottom three quintiles (medium, low and lowest) (nodes 2 and 9; p<0.001). While the splitting stopped at leaf nodes 10 and 11 for illiterate participants, it continued for the branch of literate participants. The model split the branch of literate participants belonging to higher wealth quintiles by religion (node 3) (p = 0.049), and the branch of literate participants belonging to lower wealth quintile by age at marriage (node 6)—age at marriage < = 16 vs age at marriage > 16 (p = 0.01). The splitting stopped at nodes 4, 5, 7 and 8.

The model shows that 84.0% (n = 1,229) of non-Muslim participants belonging to higher wealth quintiles (high or highest) delivered at a health facility (node 4) as compared to 76.3% (n = 194) of their Muslim counterparts (node 5). The model also documents that, for literate women who belonged to lower wealth quintiles, the outcome varied again by age at marriage—61.8% of such women (literate but poor) married at or before 16 years of age delivered at facilities vs 73.8% of literate but poor women married later than 16 years of age reporting to have delivered at facilities (nodes 7 and 8). Lastly, 68.9% of illiterate women belonging to higher wealth quintiles delivered at health facilities against 59.6% of illiterate women belonging to the lower wealth quintiles (nodes 10 and 11).

Overall, the largest difference in proportion of women delivered at a health facility was found between leaf node 4, representing non-Muslim, literate women belonging to the top 2 wealth quintiles (84.0%) vs leaf node 11, representing illiterate women belonging to the bottom three wealth quintiles (59.6%).

## Discussion

In this paper, we have explored the use of decision trees in quantifying intersections of determinants of inequities in healthcare utilization. This paper contributes to the global discourse on interactions between determinants of inequities and demonstrates the utility of an additional empirical tool to examine intersections quantitatively. The Classification and Regression Tree (CART) analysis used in the paper provides methodological advantages over the traditional approaches that have been used by researchers exploring healthcare utilization patterns among different segments of populations. Research on population segments vulnerable to low healthcare utilization has primarily used bivariate analysis of dependent variables across individual strata (e.g. caste, religion etc.) and logistic regression models assessing associations between outcomes of interest and key social determinants while controlling for other factors. Both of these methods have serious limitations in providing a deeper understanding of the intersections of socio-economic determinants of healthcare utilization. While bivariate analysis does not allow for consideration of multiple co-occurring determinants, regression models fail to specify population segments most at risk, information critical for program and policy guidance [22]. The use of CART mitigates some of these challenges by allowing consideration of multiple forms of inequity to segment populations into clearly defined sub-groups that differ based on a specified outcome, information with clear relevance for programs that intend to reduce inequities in healthcare utilization or similar resource.

Analyzing data via the CART method, we have highlighted the interplay between these determinants and demonstrated how they come together to affect healthcare utilization. Findings from our study demonstrate that interactions between household wealth, literacy and religion are significantly associated with healthcare utilization of women.

Interactions between literacy and household wealth were identified as crucial pivots in utilization of all three forms of healthcare assessed. Wealth was found to be the root node in the first two models, while literacy was the root node in the third model. The model for antenatal care checkups received in the third trimester describes the effect of this interaction most clearly. Third trimester ANC coverage among literate women in the highest wealth quintile was 18 percent higher than the average for the overall sample, whereas it was 7 percent lower among illiterate women in the bottom four wealth quintiles, thereby creating a difference of 25 percent between the two sub-groups. A similar effect was observed in the third model for women delivering at health facilities. The model found that institutional delivery among literate, non-Hindu women belonging to the top two wealth quintiles was 15 percent higher than the average for the overall sample, whereas it was 9 percent lower among illiterate women belonging to the bottom three wealth quintiles, thereby creating a difference of 24 percent between the two sub-groups.

Interestingly, wealth and literacy were found to neutralize each other’s effect on utilization. For example, in the model for pregnancy registration, literate women belonging to the bottom two wealth quintiles were found to have registered their pregnancies in the same proportion as that in the overall sample (see model 1). Similarly, wealth and literacy were found to nullify each other in relation to ANC received by women in the third trimester, with illiterate women in the highest wealth quintile and literate women in the lower wealth quintiles found to have similar levels of ANC utilization. The same finding was observed for delivery in health facilities, where there was a marginal difference in the proportion of illiterate women in the top two wealth quintiles opting for institutional delivery and that for the overall sample.

Interactions between wealth, literacy and religion were also observed. In the first model, the proportion of Muslim, illiterate women belonging to the bottom two wealth quintiles who registered their pregnancy was 15 percent lower than that of the overall sample. The interactions are further highlighted in the third model which shows that the proportion of non-Muslim, literate women belonging to the top two wealth quintiles who opted for institutional delivery was 15 percent higher than the overall sample.

Woman’s age at marriage was also found to interact with wealth and literacy in the model for delivery at health facilities, providing interesting insights. Literate women belonging to the bottom three wealth quintiles were split into two groups by age at marriage. Proportion of literate and poor women who were married very young (less than or equal to 16 years) and opted for institutional delivery was 7 percent lower than the proportion of institutional delivery in the overall sample of women. In contrast, the proportion of literate and poor women who were married after 16 years of age and opted for institutional delivery was 5 percent higher than the larger sample, illustrating an overall difference in institutional delivery of 12 percent between these two groups.

Additionally, social group and religion were also found to interact with wealth in the first model developed for pregnancy registration, however, the effect of that interaction was not found to be as strong as the effects of the interactions described above. For example, the proportion of Muslim women belonging to marginalized social groups but in the top 3 wealth quintiles was exactly the same as the proportion of the overall sample in terms of pregnancy registration. This might suggest that wealth compensates for inequities based on membership in marginalized social groups and religion. It is also interesting to note that age was not picked up as a significant element of intersection when fed into the model with other determinants.

The findings suggest that the combination of wealth and literacy plays a significant role in affecting healthcare utilization. Wealth and literacy seem to work together to increase or decrease the chances of women utilizing healthcare services. While women belonging to wealthier households do show a higher tendency to utilize healthcare, the propensity is further elevated when women are literate. Similarly, being illiterate further reduces the chances of healthcare utilization among women belonging to poorer households. Wealth and literacy also seem to neutralize the negative effects of the other, suggesting that they are similar in strength of effect on utilization of these health services. Religion was also found to interact with wealth and literacy to significantly affect utilization. It was also found that religion when working against the combination of wealth and literacy also displayed similar balancing properties as observed when wealth and literacy worked against each other in an interaction. Age at marriage was also found to significantly affect utilization when intersecting with wealth and literacy in case of institutional delivery. Significant differences were observed in the proportion of women opting for institutional delivery between women married very young (< = 16) and women married at a later age (>16) in the same sub-population of women. This suggests that women who marry at young ages may have lower personal agency regarding healthcare. In addition, social group of the respondent was also found to interact with wealth and religion, although the effect of such interactions were not found to be as strong as some of the other interactions that were explored. One of the reasons for this could be that the combined effect of wealth and religion overwhelmed the effect of marginalization based on social group. However, as noted above, the relative strength of each of the determinants in the model is difficult to assess based on the current analyses, and should be prioritized in future research on this issue.

Findings from this study further the existing understanding of intersections of social determinants of health care utilization in Uttar Pradesh, India, extending the findings of previous studies that have identified the effects of gender, literacy, social group, age at marriage and place of residence on healthcare utilizations in India [26, 27]. Sen et. al. have also demonstrated how gender and class operate together to affect non-treatment of illnesses and discontinuation of treatment [14]. The current study utilized a novel method, CART, to quantitatively clarify inequities in health care utilization among demographic segments of women of rural Uttar Pradesh based on the intersections of their wealth, literacy, social group, religion and age at marriage.

## Program implications

The models identify household wealth and literacy as the most prominent determinants whose intersections affect healthcare utilization for all the three indicators. Religion and age at marriage have also been highlighted as determinants leading to crucial intersections for utilization.

These insights provide important guidance to develop strategies to address inequities in healthcare utilization. An understanding of intersections can help programs to develop targeted interventions to bridge the inequity gap. Possible strategies include the development of tools and job-aids for health workers to help identify women belonging to specific most-at-risk segments based on their socio-demographic characteristics so at to target these individuals for interventions found to improve utilization. An example of such a tool is an indexing register that can be used by community level health workers to list all women and households in their catchment areas. Such indexing registers can capture details of the households and individuals including basic amenities in the household to assess household wealth, the educational status of women and their family members and other details like religion, social group, parity etc. For example, health workers could use these data to prioritize illiterate women belonging to poorer households to increase utilization of antenatal care and facility-based delivery.

A deeper understanding of such intersections can greatly benefit implementers and policy makers in improving coverage and utilization for the most marginalized, thereby moving towards a more equitable utilization of healthcare services.

## Limitations

One of the limitations of the CART model is that it does not provide an estimate of the relative strength of the determinants in an interaction. For example, questions like whether wealth is more potent than literacy in determining utilization remain unanswered through the use of this models.

Another limitation of using the CART algorithm to understand such intersections is that the model needs a sufficiently large sample size to undertake sub-group identification. The CART algorithm may also continue to split the sample until the sub-groups are extremely small, reducing the relevance of the inequities detected for program guidance. This is often observed in cases where no stopping criterion is provided to the model, illustrating the importance of specifying this value.

The current study also includes an analytical bias in that the categories of the independent variables utilized by the models were pre-defined by the authors. Altering the categories of the independent variables might alter the cut-off points that CART selects to split nodes into sub-groups.

Lastly, the models developed in the paper could not include an indicator of distance to health facilities (i.e., spatial inequity) or of knowledge required to access healthcare. Given the heterogeneities in access to healthcare facilities in rural Uttar Pradesh, spatial differentials are likely to intersect with social group, wealth and literacy to determine utilization. Further research is needed to understand the interplay between location and socio-economic determinants in affecting inequities in health service utilization.

## Conclusion

Despite the limitations, our analysis provides important insights into intersections of key social determinants in predicting healthcare utilization. The paper reveals the role of interactions between wealth and literacy in creating substantial disparities in healthcare utilization among women living in rural Uttar Pradesh. We also found that religion and age at marriage also interact with wealth and literacy to exacerbate disparities. The understanding of such interactions is still evolving and further research across multiple populations and geographies is required to determine its broader application. Future research should assess the utility of exploring intersections as an approach to understand other developmental issues (e.g., completion of education). Finally, although it remains to be seen how these insights may be successfully utilized from a programmatic perspective, programmatic responses to the intersections of multiple social determinants and persistent health inequities are clearly warranted.

## Ethics approval

Institutional review board approval for this study was granted from Public Health Service—Ethical Review Board (PHS-ERB) and from the Health Ministry Screening Committee (HMSC) facilitated by Indian Council for Medical Research (ICMR). IRB review and approval for the current analyses was obtained from University of California, San Diego.

## Abbreviations

ANC: Antenatal Care
CART: Classification and Regression Tree
HMSC: Health Ministry Screening Committee
ICMR: Indian Council for Medical Research
IRB: Institutional Review Board
MDGs: Millennium Development Goals
MMR: Maternal Mortality Ratio
NRHM: National Rural Health Mission
OBC: Other Backward Classes
PHS-ERB: Public Health Service- Ethical Review Board
PSU: Primary Sampling Units
SC: Scheduled Caste
SD: Standard Deviation
SDGs: Sustainable Development Goals
ST: Scheduled Tribe
TSU: Technical Support Unit
UP: Uttar Pradesh
